# Is a Hospital Pageant Practicable?

**Published:** 1921-05-21

**Authors:** Charles Cutting

**Affiliations:** Secretary. The Royal Hospital and Home for Incurables, Putney.


### IS A HOSPITAL PAGEANT PRACTICABLE?

*To the Editor of* The Hospital.

Sir,—Personally I should much regret to see a Hospital Pageant in London, where there are enough Barnum tricks already. Are there no quieter and subtler means possible? The big drum on the village green doesn't matter, but here in town it would only attract pennies and add to the general din and vulgarity and lack of order and discipline now far too common.—Yours truly,

Charles Cutting, Secretary.
The Royal Hospital and Home for Incurables, Putney.

